# Impact of training and case manager support for traditional birth attendants in the linkage of care among HIV-positive pregnant women in Southwest Nigeria: a 3-arm cluster randomized control trial

**DOI:** 10.1186/s12884-024-06332-2

**Published:** 2024-02-21

**Authors:** Adedoyin O. Ogunyemi, Kofoworola A. Odeyemi, Babasola O. Okusanya, Gbenga Olorunfemi, Melissa Simon, Mobolanle R. Balogun, Alani S. Akanmu

**Affiliations:** 1 Department of Community Health and Primary Care, College of Medicine, University of Lagos, Lagos, Nigeria; 2 Department of Obstetrics and Gyneacology, College of Medicine, University of Lagos, Lagos, Nigeria; 3 Division of Epidemiology and Biostatistics, Faculty of Health Sciences, University of Witwatersrand, Johannesburg, South Africa; 4 Department of Obstetrics and Gynaecology, Robert H. Lurie Comprehensive Cancer Center, Northwestern University Feinberg School of Medicine, Chicago, USA; 5 Department of Haematology and Blood Transfusion, College of Medicine, University of Lagos, Lagos State, Lagos, Nigeria

**Keywords:** Case managers, PMTCT, HIV, Nigeria, Traditional birth attendants, Training

## Abstract

**Background:**

Mother-to-child transmission (MTCT) accounts for 90% of all new paediatric HIV infections in Nigeria and for approximately 30% of the global burden. This study aimed to determine the effectiveness of a training model that incorporated case managers working closely with traditional birth attendants (TBAs) to ensure linkage to care for HIV-positive pregnant women.

**Methods:**

This study was a 3-arm parallel design cluster randomized controlled trial in Ifo and Ado-Odo Ota, Ogun State, Nigeria. The study employed a random sampling technique to allocate three distinct TBA associations as clusters. Cluster 1 received training exclusively; Cluster 2 underwent training in addition to the utilization of case managers, and Cluster 3 served as a control group. In total, 240 TBAs were enrolled in the study, with 80 participants in each of the intervention and control groups. and were followed up for a duration of 6 months. We employed a one-way analysis of variance (ANOVA) statistical test to evaluate the differences between baseline and endline HIV knowledge scores and PMTCT practices. Additionally, bivariate analysis using the chi-square test was used to investigate linkage to care. Furthermore, logistic regression analysis was utilized to identify TBA characteristics associated with various PMTCT interventions, including the receipt of HIV test results and repeat testing at term for HIV-negative pregnant women. The data analysis was performed using Stata version 16.1.877, and we considered results statistically significant when *p* values were less than 0.05.

**Results:**

At the end of this study, there were improvements in the TBAs’ HIV and PMTCT-related knowledge within the intervention groups, however, it did not reach statistical significance (*p* > 0.05). The referral of pregnant clients for HIV testing was highest (93.5%) within cluster 2 TBAs, who received both PMTCT training and case manager support (*p* ≤ 0.001). The likelihood of HIV-negative pregnant women at term repeating an HIV test was approximately 4.1 times higher when referred by TBAs in cluster 1 (AOR = 4.14; 95% CI [2.82–5.99]) compared to those in the control group and 1.9 times in cluster 2 (AOR = 1.93; 95% CI [1.3–2.89]) compared to the control group. Additionally, older TBAs (OR = 1.62; 95% CI [1.26–2.1]) and TBAs with more years of experience in their practice (OR = 1.45; 95% CI [1.09–1.93]) were more likely to encourage retesting among HIV-negative women at term.

**Conclusions:**

The combination of case managers and PMTCT training was more effective than training alone for TBAs in facilitating the linkage to care of HIV-positive pregnant women, although this effect did not reach statistical significance. Larger-scale studies to further investigate the benefits of case manager support in facilitating the linkage to care for PMTCT of HIV are recommended.

**Trial registration:**

The study was retrospectively registered in the Pan African Clinical Trial Registry, and it was assigned the unique identification number PACTR202206622552114.

**Supplementary Information:**

The online version contains supplementary material available at 10.1186/s12884-024-06332-2.

## Background

The use of traditional birth attendants (TBAs) in antenatal services and delivery is common in Nigeria, and up to 20–60% of births occur in the facilities of TBAs, especially in rural Nigeria [1, 2]. The World Health Organization (WHO) defines a TBA as “a person who assists the mother during childbirth and initially acquired her skills by delivering babies herself or through an apprenticeship to other TBAs” [3]. Despite the essential role TBAs play in the health system, they are perceived to contribute to the high maternal mortality ratio in Nigeria due to some adverse practices and possibly inadequate knowledge [4].

Mother-to-child transmission (MTCT) accounts for 90% of all new paediatric human immunodeficiency virus (HIV) infections in Nigeria and for approximately 30% of the global burden [5, 6]. In developed countries, evidence-based interventions have been introduced to reduce the MTCT of HIV rates to < 2%, yet high transmission rates persist in sub-Saharan Africa, possibly due to low rates of access to HIV testing (11.7%) and antiretroviral prophylaxis (17.1%) [7, 8]. Although only 39% of all deliveries take place in the health facility in Nigeria, facility-based antenatal care is still the main vehicle used for PMTCT services in Nigeria [1]. Therefore, it is essential to create interventions to improve non-facility prevention of MTCT of HIV [9, 10].

Previous studies have reported that the involvement of TBAs in HIV prevention and PMTCT programs can reduce new paediatric HIV infections since the majority of paediatric HIV infections in our environment are due to vertical transmission [10–12]. Such TBA engagements include training on HIV counselling and testing and on some PMTCT services, including administering nevirapine syrups to newborns [13]. The challenge with TBAs’ involvement in the PMTCT service cascade includes poor linkage to HIV care and inappropriate or non-utilization of current PMTCT guidelines to manage the identified HIV-positive pregnant woman and their exposed infants [14, 15]. To close the ‘linkage to care’ gap, community support services such as the utilization of case managers have been successful in community antiretroviral services [16, 17]. The case manager makes referrals, coordinates care with facility providers, and manages the exchange of information between providers and human services organizations. This aligns with target two of the Sustainable Development Goal three (SDG 3, target two), which is to end preventable deaths of newborns and children under 5 years of age [18, 19].

According to the WHO, coordination of care is a global priority for reorienting health services to the needs of people [20]. Some studies have demonstrated the successful implementation of training and support from care coordinators in improving medication adherence among individuals with HIV and keeping them engaged and connected to care [21, 22]. However, more research is needed to identify the most efficacious interventions in settings with limited resources. This study compared the effectiveness of usual care (control arm), PMTCT training of TBAs alone versus a combination of training and community support service providers (case managers) to ensure PMTCT care and linkage to PMTCT centres for HIV-positive pregnant women.

## Methods

### Trial design

This study employed a 3-arm parallel design cluster randomized controlled trial to assess and compare the effectiveness of two interventions. The first arm comprised TBAs who received PMTCT training only (Cluster 1), and the second arm included TBAs who received both PMTCT training and case manager support (Cluster 2). The third group served as the control group and received neither training nor case manager support (Control). The participant allocation ratio across the three arms was 1:1:1.

### Study location

The study locations were Ifo and Ado-Odo/Ota Local Government Areas (LGAs) in Ogun State, Southwest Nigeria. Ado-Odo/Ota is the largest LGA out of the 20 LGAs in Ogun State, with an area of 234,647 km^2^, and it is popular for traditional, cultural and historic festivals, with a population of 526,565 [23]. Ifo LGA is a neighbouring LGA to Ado-Odo/Ota and has an area of 215,055km^2^ and a population of 524,837 [24]. These two LGAs serve as a rural health setting for training doctors and medical students at the College of Medicine, University of Lagos. The 2 LGAs are approximately 46 km apart from each other.

### Study population and selection of participants

The TBAs in Ifo and Ado-Odo/Ota LGAs belong to three different associations and have separate meeting times and venues. The associations are registered with the LGA, which provides some regulation to their activities. To limit contamination among the groups, the controls were recruited from Ifo LGA, while the training-only and training plus utilization of case manager groups were from Ado Odo/Ota LGA. These associations are clustered and consist of approximately 100 members each having similar demographic and socioeconomic characteristics but are situated in different geographic wards. The eligibility criteria included registered TBAs aged 18 years or older who recorded two or more births in their birthing homes in the previous month, had proper record documentation and had not received PMTCT training previously.

The research team worked with the medical officer of health (MOH), head of TBA associations and LGA staff in determining TBAs who met the eligibility criteria. After the list had been compiled, all eligible TBAs were invited to participate in the study by sending text messages to their phones. Sixteen staff from the LGA with some health training background were selected and trained to assist with data collection. Twelve of them served as research assistants, and four were trained as case managers (CM). Four research assistants were assigned to the control group, another four research assistants to cluster 1, and four research assistants and four case managers to cluster 2. In each cluster meeting in a central hall at the respective LGAs, the research assistants screened all participants to identify eligible respondents. Subsequently, they were given information about the study and administered consent forms (in English and Yoruba languages) for willing participants to sign as consent to be involved in the study.

### Sample size determination

The sample size formula for randomized controlled trials was used to calculate the sample size for each group to achieve a power of 80%, an effect size of 15% and a statistical significance of 0.05 (2-sided) in a superiority trial given that the prevalence of the practice of PMTCT strategies among TBAs was 43.0% from a previous study [25]. A minimum sample size of 42 TBAs per arm was needed, but this was doubled to 80 TBAs per arm totalling 240 TBAs to allow for robust regression modelling.

### Randomization and allocation

After the baseline assessment of the participants, a representative of each of the 3 TBA clusters was invited by the principal investigator to a meeting. At the meeting, each TBA representative picked an opaque sealed envelope from a box that contained an allocation card that indicated the study arm to which the participating TBAs in her association would be assigned. One cluster was randomized to PMTCT training only, another cluster to the training and case managers, while the third cluster of TBAs served as controls that neither had training nor case managers assigned to it.

Meetings were held with TBAs in each of the three groups in equal proportions (80 each), and a research assistant was assigned to each group. The research assistants introduced themselves to the TBAs individually, collected the TBA birthing home details and distributed TBA logs to each TBA. The case managers were assigned to TBAs in cluster 2 only. Additionally, the CMs gave their contact details on how they could be reached by the TBA for ease of contact if they identified a pregnant woman who tested HIV-positive. The TBAs in the training and control clusters were also given the details of six PMTCT centres within the LGAs where referrals should be made, while cluster 2 was asked to contact their case managers in case a pregnant positive woman was identified. Furthermore, an independent research assistant verified the successful referral process by ensuring the coloured plastic bottle covers, initially provided to the TBAs, for the use of HIV-positive pregnant women, were handed over to the laboratory head at any of the designated PMTCT centres. In cluster 2, the TBA informed the case manager when a pregnant woman tested HIV positive. The case manager contacted the HIV-positive pregnant woman and accompanied her to the designated PMTCT centre.

### Interventions

The TBAs in cluster 1 received PMTCT training only. This included didactic modules that took place for 2 days. It included topics on the natural history of HIV, antenatal testing, methods of MTCT of HIV, cART (combination antiretroviral therapy) in pregnancy and infant testing. Furthermore, information on PMTCT referral centres within the LGAs, patient confidentiality and the use of the TBA log register (S1 Appendix) were given. Cluster 2 similarly received the above training and case manager support. The case manager was assigned to the TBA and visited the TBAs in their homes monthly to review the TBA logs. Additionally, the TBA was to inform the case manager if any pregnant woman under their care tested HIV-positive. The case manager would then facilitate linkage support by taking the HIV-positive pregnant woman in cluster 2 to predetermined PMTCT sites within the LGA. The training was conducted in the local language (Yoruba). All three groups were assigned research assistants who reported data from the TBA logs at the end of each month for the period of 6 months.

All TBAs who participated in the study were provided with delivery kits as incentives for their clients. The components of the kit were meant to promote WHO hygienic birth practices, clean delivery and cord care [26]. At baseline and at the end of the study period, PMTCT knowledge and practices were assessed for all TBAs using a pretested, interviewer-administered structured questionnaire in the Yoruba language. TBA logs contained the steps in the linkage cascade according to the WHO guidelines [27–29].

## Trial outcomes

### Primary outcomes

The proportion of women who were linked to care at the PMTCT centre (i.e HIV-positive pregnant women who present at the PMTCT centre) or a repeat HIV test at term/labour in the case of an HIV-negative pregnant woman. The focal health worker in each of the assigned PMTCT centres was visited at the end of each month during the study to determine the number of coloured plastic bottle covers deposited by the HIV-positive pregnant women who presented at any of the centres. This was thereafter verified by viewing the PMTCT register to determine the date of linkage to care of the pregnant women. The status of a repeat HIV test at term or before delivery was determined after the discharge of the HIV-negative woman by viewing the TBA register.

### Secondary outcome

The secondary outcome was to compare study arms with respect to the degree of changes in knowledge and PMTCT practices of TBAs. This was done after the end-of-study data collection.

### Training of research assistants

The research assistants and case managers received a 2-day training from an obstetrician on topics including the natural history of HIV, antenatal testing, methods of MTCT of HIV, ARV treatment in pregnancy and infant testing. Furthermore, the four case managers were also trained on strategies for linkage using the national guidelines for HIV prevention, treatment and care [30]. The 12 research assistants were assigned to TBAs in cluster 1 and cluster 2 and control groups to collect data from the TBA log registers on PMTCT activities at the end of each month for a total of 6 months. The four case managers were assigned to TBAs in cluster 2 only to support PMTCT linkage services of HIV-positive pregnant women.

## Data management

The data collection was conducted using a semi-structured interviewer-administered questionnaire adapted from a previous study on PMTCT knowledge and practices among TBAs [25]. The questionnaire consisted of specific sections to assess various aspects of knowledge and practices related to HIV and PMTCT. For the assessment of knowledge about HIV, seven questions were included. Respondents were required to select the correct answer from a list of options, distinguishing between accurate and inaccurate responses. Each correct response was assigned a score of one, while each incorrect response received a score of zero. Participants who provided four or more correct responses, including an accurate description of HIV, were categorized as having HIV knowledge.

Similarly, PMTCT knowledge was evaluated using five questions, which covered topics such as awareness of PMTCT and the modes of transmission of HIV from mother to child. Correct responses to these questions were assessed individually. To assess PMTCT practices among TBAs, specific aspects were examined, including their counselling of pregnant women and their referral of pregnant women for HIV testing. TBAs were encouraged to refer their clients to regulated laboratories for both their initial and repeat HIV testing (when negative), and only HIV test results from such referral laboratories were considered. The receipt of HIV test results was verified through documentation in the TBA registers for pregnant women. Additionally, the linkage to care of pregnant women who tested HIV-positive was confirmed by visiting the designated PMTCT centres. Furthermore, a repeat HIV test at term was defined as a test performed either after the 37th week of gestation and before the delivery process.

## Data analyses

The data were analysed using Stata IC version 16.1.877 (StataCorp, USA) statistical software. Frequencies and proportions of participants’ sociodemographic characteristics, baseline and follow-up knowledge and practices were computed. One-way analysis of variance (ANOVA) was also conducted for the association between continuous variables and the arm of the study. Univariable and multivariable binary logistic regression analyses were used to adjust for significant differences identified at baseline between the intervention and standard practice arms in the outcome analyses. Variables with a *P* value < 0.2 were utilized to build the model in a backwards elimination technique. Some variables, such as TBA age, level of education, years of practice, HIV and PMTCT knowledge, which could be associated with PMTCT practices were chosen a priori based on a previous study [25]. A two-tailed test of hypothesis was assumed, and the level of significance was set at *p* < 0.05.

## Results

### Participant flow

**Fig. 1:**
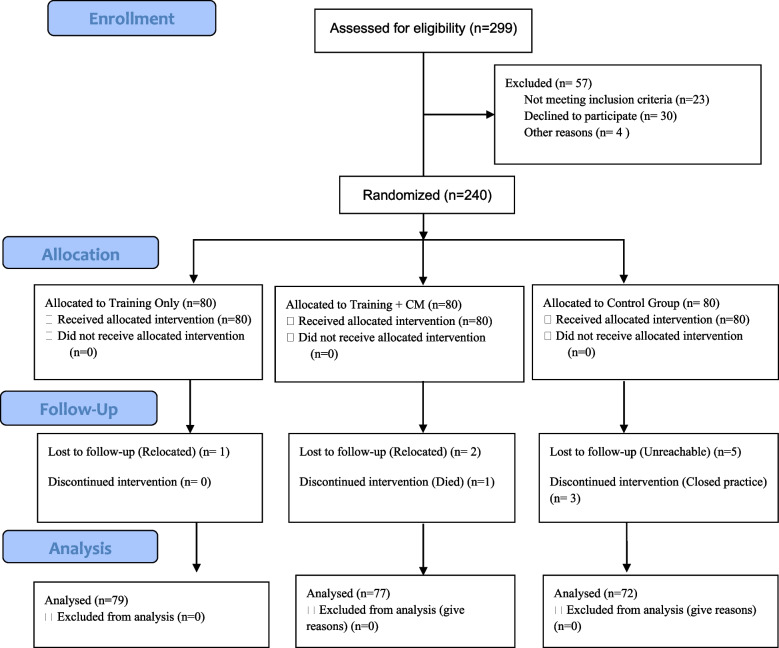
Participant flowchart for parallel design, based on CONSORT guidelines for transparent reporting of trials.^r^ CONSORT-Consolidated Standards of Reporting Trials; CM- Case Manager

The study had a total of 240 TBA participants (80 per cluster) at baseline. They were followed up for 6 months, and at the end, 12 participants (1 in cluster 1, 3 in cluster 2 and 8 in cluster 3) were lost to follow-up (see Fig. 1: Participant flowchart for parallel design, based on CONSORT guidelines for transparent reporting of trials [31]), and data from 228 TBAs were analysed at the end of the study.

### Recruitment

Study participants were recruited in September 2019, and follow-up took place for 6 months as planned between October 2019 and March 2020.

### Sociodemographic characteristics of the TBAs

The TBAs were predominantly female, with only 4 males (6.7%) in the study. There were no statistically significant differences between gender (*P* value = 0.78), educational status (*P* value = 0.18) and years of practice (*P* value = 0.42) across the three arms of the study. There was a statistically significant difference (*p* = 0.04) in the mean age across the TBA clusters, with TBAs in Cluster 1 having the highest mean age (46.6 ± 7.4 years) and the longest duration of training as a TBA in Cluster 2 (*P* value = 0.01) (Table 1).

**Table 1:** Baseline demographic characteristics of the TBAs in the 3 clusters (*n* = 240)

Variables	Cluster 1 (Training only) *n* = 80	Cluster 2 (Training + Case Managers) *n* = 80	Control *n* = 80	F	*p* value
**Gender**					
Female	79 (98.7)	78 (97.5)	79 (98.7)	0.25	0.78
Male	1 (1.3)	2 (2.5)	1 (1.3)		
**Age in years**					
(Mean ± SD)	46.4 (7.4)	41.7 (8.3)	44.8 (9.6)	5.96	0.04*
**Education**					
None	0 (0.0%)	1 (1.2%)	0 (0.0%)	3.31	0.04*
Primary	4 (5.0%)	5 (6.3%)	4 (5.0%)		
Secondary	44 (55.0%)	57 (71.2%)	54 (67.5%)		
Post-Secondary	32 (40.0%)	17 (21.3%)	22 (27.5%)		
**Mean years of Practice**					
(±SD)	16.4 (9.4)	12.7 (7.5)	15.5(9.4)	3.75	0.42
**Training duration as a TBA**					
(Years)	3.2 (1.1)	3.7 (1.6)	3.0(1.2)	6.09	0.01*
**Trainer**					
Parent	1 (1.7%)	3 (3.7%)	5(6.3%)	1.38	< 0.001*
Relative	2 (3.3%)	6 (7.5%)	3 (3.7%)		
TBA Expert	18 (30.0%)	45 (56.3%)	30 (37.5%)		
Others	60 (75.0%)	26 (32.5%)	44 (55.0%)		
**Total number of pregnant women receiving ANC**	876	712	434		

*p-value <0.05

### Baseline knowledge and practices regarding HIV and PMTCT of TBAs

At baseline, 64 (80.0%) participants in cluster 1, 65 (81.2%) in cluster 2 and 61 (76.2%) in the control group had correct knowledge of HIV as an infection (*P* value = 0.72). The lowest proportion was among the control group, but these differences were not statistically significant (*P* value = 0.72). Similarly, there were no statistically significant differences in the participants’ PMTCT awareness (*P* value =0.25), transmission of HIV from mother to child at different stages of pregnancy (*P* value = 0.93), delivery (*P* value = 0.43) or breastfeeding (*P* value =0.42). Approximately a quarter of all TBAs in each of the groups reported that they could conduct the HIV tests by themselves, and about half of the TBAs in each of the clusters kept records of the HIV status of their clients (Table 2).

**Table 2:** Baseline knowledge and reported practices regarding HIV and PMTCT in the 3 clusters (*n* = 240)

Knowledge and Practices (Yes/No)	Cluster 1 *n* = 80 (%)	Cluster 2 *n* = 80 (%)	Control *n* = 80 (%)	F	*P*-value
Correct knowledge of HIV	64 (80.0)	65 (81.2)	61 (76.2)	0.33	0.72
Aware of PMTCT of HIV	74 (92.5)	68 (85.0)	68 (85.0)	1.37	0.25
MTC of HIV is possible	76 (95.0)	76 (95.0)	73 (91.2)	0.64	0.53
MTCT of HIV can occur in pregnancy	48 (60.0)	48 (60.0)	46 (57.5)	0.07	0.93
MTCT of HIV can occur at delivery	38 (47.5)	35 (43.7)	30 (37.5)	0.83	0.43
MTCT of HIV can occur while breastfeeding	44 (55.0)	46 (57.5)	38 (47.5)	0.87	0.42
HTS can prevent MTCT of HIV	45 (56.2)	38 (47.5)	40 (50.0)	1.06	0.34
TBA can conduct HIV test	18 (22.5)	20 (25.0)	20 (25.0)	0.09	0.91
**Refer for HIV test**					
Yes, for all	70 (87.5)	67 (83.7)	69 (86.3)	0.29	0.91
Yes, for some	7 (8.6)	8 (10.0)	6 (7.5)		
Not at all	3 (3.7)	5 (6.3)	5 (6.2)		
**Know HIV status of your pregnant clients**					
Yes, for all	75 (93.7)	71 (88.8)	76 (95.0)	0.85	0.47
Yes, for some	3 (3.8)	5 (6.2)	1 (1.3)		
Not at all	2 (2.5)	4 (5.0)	3 (3.7)		
**Records for HIV Status**	51 (63.7)	45 (56.2)	46 (57.5)	0.53	0.59

### End-of-study knowledge and practices regarding HIV and PMTCT of TBAs

At the end of the study, only 228 respondents (79 in cluster 1, 77 in cluster 2 and 72 in the control group) were available to be interviewed (Fig. 1). Correct knowledge about HIV was 100% in clusters 1 and 2 and 98.6% in cluster 3, but this difference was not statistically significant. The knowledge that MTCT of HIV can occur during delivery was highest in TBA respondents in cluster 1 (86.0%) and this difference was statistically significant (*p* = 0.04). Similarly, participants who referred all their pregnant women for HIV testing was highest among cluster 2 TBAs (*p* < 0.001). Among the TBA respondents who knew the HIV status of their pregnant women, it was highest (98.6%) in the control group and lowest (94.8%) in cluster 2 but this difference did not achieve statistical significance (*p* = 0.07) (Table 3).

**Table 3:** End-of-study knowledge and reported practices regarding HIV and PMTCT in the 3 clusters

Knowledge and Practices (Yes/No)	Cluster 1 *n* = 79	Cluster 2 *n* = 77	Control *n* = 72	F	*p*-value
Correct knowledge of HIV	79 (100.0)	77 (100.0)	71 (98.6)	1.08	0.34
Aware of PMTCT	71 (89.9)	71 (92.2)	67 (93.1)	0.27	0.76
MTCT of HIV is possible	76 (96.2)	74 (96.1)	70 (97.2)	0.08	0.92
MTCT can occur in pregnancy	45 (57.0)	42 (54.5)	50 (69.4)	1.98	0.14
MTCT can occur at delivery	68 (86.0)	53 (68.8)	55 (76.4)	3.37	0.04*
MTCT of HIV can occur while breastfeeding	59 (74.7)	63 (81.8)	64 (88.9)	2.55	0.08
HTS can prevent MTCT	47 (59.5)	67 (87.0)	66 (91.7)	15.76	< 0.001*
TBA can conduct HIV test	36 (46.1)	19 (24.6)	15 (20.8)	6.99	0.001*
**Refer for HIV test**					
Yes, for all	53 (67.1)	72 (93.5)	66 (91.7)	9.58	< 0.001*
Yes, for some	25 (31.6)	2 (2.6)	3 (4.2)		
Not at all	0 (0.0)	0 (0.9)	3 (4.2)		
**Know HIV status of your pregnant clients**					
Yes, for all	75 (96.2)	73 (94.8)	71 (98.6)	5.07	0.07
Yes, for some	3 (3.8)	4 (5.2)	1 (1.4)		

*p-value <0.05

### TBA’s PMTCT practices in pregnant women

The data from the TBA log sheets were collected once a month. For a period of 6 months, a total of 2022 pregnant women registered with TBAs. Cluster 2 TBAs referred 99.4% of their clients for HIV tests, which was higher than in the cluster 1 (94.4%) and control (94.4%) groups, and this difference was statistically significant (*p* < 0.001). In cluster 1, 31% of TBAs referred pregnant women for HIV tests at term (between 37 weeks of gestation and before delivery) if they had initially tested negative during booking.” This was higher than that among TBAs in cluster 2 (15.8%) and the control group (9.7%), and this difference was statistically significant (*P* value < 0.001) (Table 4).

**Table 4:** End-of-study data of the TBA’s PMTCT practices among registered pregnant women (over 6 months)

PMTCT data of pregnant women registered for ANC by TBAs	Cluster 1 *n* = 876 (%)	Cluster 2 *n* = 712 (%)	Control *n* = 434 (%)	F	*P* value
Referral for HIV test at any time during pregnancy (before term or delivery)	826 (94.4)	707 (99.4)	410 (94.4)	15.6	< 0.001*
Referral for HIV test at first visit	556 (67.6)	400 (56.7)	287 (69.7)	13.4	< 0.001*
Test results received	810 (97.9)	688 (97.2)	397 (96.3)	1.38	0.25
Test Result					
HIV Negative	804 (99.3)	683 (99.3)	393 (99.0)	0.15	0.86
HIV Positive	6 (0.74)	5 (0.73)	4 (1.01)		
HIV test repeated at term or before delivery (for initial negative test)	249 (30.9)	108 (15.8)	38 (9.7)	46.0	< 0.001*

*p-value <0.05

### Association between TBA characteristics and linkage to care in HIV-positive pregnant women

For factors associated with the linkage to care of HIV-positive pregnant women to the PMTCT centres, all 5 (100.0%) of the HIV-positive pregnant women in cluster 2 were linked to the PMTCT centre compared to 4 (66.7%) in cluster 1 and 1 (25.0%) in the control group. However, this difference was not statistically significant (χ^2^ = 5.63, *p* = 0.06). Other characteristics, such as the TBA’s age, level of education, years of practice and knowledge of HIV, were not significantly associated with the linkage of care for HIV-positive pregnant women (*p* > 0.05) (Table 5).

**Table 5:** Comparison between TBA characteristics and linkage to care among HIV-positive pregnant women

Variables	Linkage to PMTCT centre (*n* = 15)		χ^2^	*p* value
	No (*n* = 5)	Yes (*n* = 10)		
**Arms**				
Control	3 (75.0)	1 (25.0)	5.63	0.06
Cluster 1 (Training)	2 (33.3)	4 (66.7)		
Cluster 2 (Training + CM)	0 (0.0)	5 (100.0)		
**Age group (years)**				
20–39	1 (50.0)	1 (50.0)	2.63	0.269
40–59	3 (25.0)	9 (75.0)		
> 60 years	1 (100.0)	0 (0.0)		
**Level of Education**				
Primary	1 (100.0)	0 (0.0)	3.3	0.192
Secondary	2 (20.0)	8 (80.0)		
Post- Secondary	2 (50.0)	2 (50.0)		
**Years of practice**				
1–10	2 (50.0)	2 (50.0)	3.75	0.153
11–20	3 (50.0)	3 (50.0)		
Above 20	0 (0.0)	5 (100.0)		
**Referral for HIV test at first visit**				
No	4 (50.0)	4 (50.0)		
Yes	1 (14.3)	6 (85.7)	2.15	0.282
**Correct knowledge of HIV at baseline**				
No	1 (50.0)	1 (50.0)	0.28	0.571
Yes	4 (30.8)	9 (69.2)		

### Factors associated with the receipt of HIV test results of pregnant women

In the logistic regression analysis, low education (primary) had an odds ratio of 0.21 (95%[CI]: [0.08, 0.5]), indicating a decreased likelihood of the receipt of HIV test results and this was statistically significant (*p* < 0.05). None of the other TBA characteristics demonstrated statistical significance (*p* > 0.05) in predicting the likelihood of receiving the HIV test results of the pregnant women in their care (Table 6).

**Table 6:** Logistic regression of factors associated with the receipt of HIV test results of pregnant women

Variables	Unadjusted OR (95% CI)	*ρ* value	Adjusted OR (95% CI)	*ρ* value
**Arms**				
Control	1.00		1.00	
Cluster 1 (Training)	1.80 (0.9–3.63)	0.102	1.73 (0.84–3.6)	0.137
Cluster 2 (Training + CM)	1.3 (0.66–2.56)	0.450	1.28 (0.62–2.66)	0.495
**Age group (years)**				
20–39	1.00		1.00	
40–59	5.99 (0.61–1.97)	0.764	1.30 (0.64–2.66)	0.463
Above 60	1.68 (0.22–12.93)	0.616	11.2 (1.2–105.6)	0.033*
**Level of Education**				
None	1.00		1.00	
Primary	0.21 (0.08–0.5)	0.001*	0.11 (0.04–0.29)	< 0.001*
Secondary	1.68 (0.39–1.4)	0.394	0.77 (0.4–2.12)	0.444
**Years of practice**				
1–10	1.00		1.00	
11–20	0.57 (0.57–1.99)	0.859	1.01 (0.5–2.03)	0.979
Above 20	1.06 (0.52–2.18)	0.864	0.35 (0.38–2.12)	0.810
**Correct knowledge of HIV at baseline**				
No	1.00		1.00	
Yes	0.58 (0.31–2.69)	0.098	0.43 (0.17–1.13)	0.085

*Statistically significant

### Factors associated with repeat testing at term for HIV-negative pregnant women

The likelihood of HIV-negative pregnant women at term repeating an HIV test was approximately 4.1 times higher when referred by TBAs in cluster 1 (AOR = 4.14; 95% CI [2.82–5.99]) compared to those in the control group and 1.9 times in cluster 2 (AOR = 1.93; 95% CI [1.3–2.89]) compared to the control group. Additionally, older TBAs (OR = 1.62; 95% CI [1.26–2.1]) and TBAs with more years of experience in their practice (OR = 1.45; 95% CI [1.09–1.93]) were more likely to encourage retesting among HIV-negative women at term (Table 7).

**Table 7:** Logistic regression of factors associated with repeat testing at term for HIV-negative pregnant women

Variables	Unadjusted OR (95% CI)	*ρ* value	Adjusted OR (95% CI)	*ρ* value
**Arms**				
Control	1.00		1.00	
Cluster 1 (Training)	4.1 (2.83–5.93)	< 0.001*	4.14 (2.82–5.99)	< 0.001*
Cluster 2 (Training + CM)	1.73 (1.16–2.56)	0.006	1.93 (1.3–2.89)	0.001*
**Age group (years)**				
20–39	1.00		1.00	
40–59	1.62 (1.26–2.1)	< 0.001*	1.12 (1.22–1.52)	0.479
**Level of Education**				
None	1.00		1.00	
Primary	2.05 (0.25–0.94)	0.032*	0.86 (0.56–2.41)	0.692
Secondary	0.81 (0.64–1.02)	0.07	1.04 (0.40–1.49)	0.729
**Years of practice**				
1–10	1.00		1.00	
11–20	1.25 (0.96–1.6)	0.101	0.17 (0.27–1.55)	0.288
Above 20	1.45 (1.09–1.93)	0.01*	1.4 (1.01–1.95)	0.049*
**Correct knowledge of HIV at baseline**				
No	1.00		1.00	
Yes	1.08 (0.13–1.43)	0.59	1.16 (0.85–1.57)	0.342

*Statistically significant

### Factors associated with linkage to care in HIV-positive pregnant women

In the logistic regression analysis using Firth logistic regression, the likelihood for HIV-positive pregnant women in cluster 2 being linked to care was approximately 40.0 times higher when compared with the control group, (AOR = 40.0; 95% CI [0.8–820.6]), however, the result did not achieve statistical significance (*p* > 0.05). Similarly, other factors indicated no significant association between TBA characteristics and linkage to care (Table 8).

**Table 8:** Firth logistic regression of factors associated with linkage to care among HIV-positive pregnant women

Variables	Unadjusted OR (95% CI)	*ρ* value	Adjusted OR (95% CI)	*ρ* value
**Arms**				
Control	1.00		1.00	
Cluster 1 (Training)	4.22 (0.36–48.9)	0.253	1.1 (0.05–149.9)	0.627
Cluster 2 (Training + CM)	25.7 (0.80–820.6)	0.067	40.0 (0.74–1652.4)	0.052
**Age group (years)**				
20–39	1.00		1.00	
40–59	1.1 (0.21–35.5)	0.447	0.24 (0.002–26.8)	0.555
Above 60	0.33 (\0.01–16.7)	0.583	4.09 (0.004–4817.4)	0.694
**Level of Education**				
None	1.00		1.00	
Primary	10.2(0.31–337.0)	0.193	9.68 (0.08–1141.4)	0.351
Secondary	3.0 (0.08–16.8)	0.555	–	
**Years of practice**				
1–10	1.00		1.00	
11–20	4.57 (0.1–9.87)	0.998	0.20 (0.006–66.7)	0.590
Above 20	3.0 (0.08–323.8)	0.165	3.28 (0.05–212.7)	0.572
**Correct knowledge of HIV at baseline**				
No	1.00		1.00	
Yes	2.11 (0.17–26.3)	0.562	1.28 (0.004–365.0)	0.93

## Discussion

This 3-arm study assessed and compared the effectiveness of PMTCT training-only and training of TBAs along with the utilization of case managers in the prevention of mother-to-child transmission of HIV. The study aimed to evaluate interventions for identifying HIV-positive pregnant women and their linkage to PMTCT centres for care. There were 240 TBAs who rendered antenatal care services to 2022 pregnant women over a period of 6 months.

At baseline, there were no significant differences in HIV-related knowledge and PMTCT practices among the 3 groups. However, TBAs in cluster 1 (training-only) and cluster 2 (training and case manager support) demonstrated higher levels of awareness regarding the HIV status of their pregnant clients and better record-keeping practices, with these differences being statistically significant (*p* < 0.05). Previous studies have highlighted the advantages of involving trained and supervised TBAs in preventing mother-to-child transmission of HIV [10, 32, 33]. Nevertheless, by the end of this study, there were no significant improvements in the TBAs’ knowledge about how HIV can be transmitted from a mother to her child during pregnancy and breastfeeding across the 3 groups. Training TBAs in low- and middle-income countries to enhance their knowledge and practices related to PMTCT of HIV has yielded mixed results. These variations can be attributed to several challenges, including the TBAs’ lower levels of education, difficulties in reporting practices and the inadequacy of supervision quality [34]. It was observed that the effectiveness of this training was more pronounced in regions where essential obstetric care services were readily available and could be utilized as referral options by TBAs [35]. In such contexts, the indirect impact of training, which facilitated referrals to higher-level healthcare facilities when needed, appeared to be more beneficial than solely focusing on direct training for the TBAs themselves.

Our study revealed that TBAs who underwent training and had access to case managers demonstrated a remarkable rate of referring nearly all of their pregnant clients for HIV tests with 99.4% compliance. This rate was significantly higher than the proportion of HIV testing seen in the other clusters (*p* < 0.001). This aligns with findings from previous studies conducted in Nigeria, rural Zambia and Malawi [9, 10, 36], which also indicated that TBAs who received PMTCT training were more likely to refer for HIV tests, thus contributing to increased awareness of HIV status. Despite the relatively high proportion of HIV test referrals observed across all clusters, the practice of HTS referral during the initial visit was less common. The control group had the highest improvement of HTS referrals between baseline and endline assessments.

The HIV positivity rate in this study was 0.7%, which is lower than the 2019 national average of 1.4% [37]. The recent drop in estimates has been attributed to enhanced surveillance [38]. However, Nigeria has not met the UNAID’s 2015 target of the countdown to zero with the Global Plan towards the elimination of new HIV infections among children [39]. Achieving this is hinged on identifying pregnant women living with HIV and giving access to antiretroviral drugs through their linkage to care.

In our study, we observed that less than a third of the pregnant women were referred for an HIV test at term or during the process of labour. This finding is notably lower than 52.1% of pregnant women in Vietnam who received repeat HIV testing [40]. The latter study comprised women who had their babies in health facilities with well-established PMTCT programs, which likely contributed to the higher proportion of repeat testing in that context.

Furthermore, our study revealed variations in the rates of repeat HIV testing among different clusters. The training-only cluster had the highest rate at 30.9%, while the control group had the lowest rate at 9.7%. This difference was statistically significant (*p* < 0.001). Research has emphasized the importance of conducting provider-initiated counselling and testing (PICT) not only during the initial antenatal care (ANC) visit but also at term/delivery. This is because some pregnant women may seroconvert to HIV later in their pregnancy, underscoring the necessity of repeat tests [41]. HIV incidence proportions among pregnant women who initially tested negative but later seroconverted were observed at 0.41% in Kenya [41], 0.12% in India [42], 0.5% in Zambia [43]. and 3.3% in South Africa [44]. These statistics highlight the ongoing risk of HIV infection during pregnancy and the importance of repeat testing to ensure timely diagnosis and appropriate interventions.

In cluster 2, which involved training and utilization of case managers, all pregnant women who tested positive for HIV were linked to care at the designated PMTCT centres. This rate was higher compared to the training-only cluster and control groups, where 66.7 and 25.0% of HIV-positive pregnant women, respectively, demonstrated evidence of being linked to care. This suggests that the implemented strategies, especially in the cluster involving training and utilization of case managers, have contributed to a higher rate of successful linkage to PMTCT services. However, these differences were not statistically significant (*p* > 0.05%). Additionally, factors such as the TBA’s age, level of education, years of practice and knowledge of HIV did not show significant associations with the linkage to care for HIV-positive pregnant women (*p* > 0.05). This aligns with findings of other studies, which have highlighted the role of HIV case managers with appropriate training and resource support in promoting treatment adherence and improving cluster of differentiation 4 (CD4) counts among HIV patients [45, 46].

From our study, the training and use of case manager interventions did not affect receiving HIV test results from the pregnant women in the TBAs care, as almost all the pregnant women who had been requested to undertake the HIV test did so. It is acknowledged that TBAs are powerful actors in maternal health issues due to cultural beliefs, norms and practices [47]. TBAs who belonged to the intervention groups, who were older, and who had spent more years in practice were more likely to refer for a HIV retest at term to pregnant women who were initially negative. In view of the significant proportion of pregnant women in Nigeria who do not deliver at health centres, innovative ways to identify and link HIV-positive pregnant women to HIV care and treatment are critical to eliminating MTCT of HIV [48–51].

## Study limitations

There are several strengths in this study, including a large sample size of TBAs and pregnant women in their care. However, some limitations exist. One potential limitation is the close proximity of the study sites from which the TBA groups were recruited. This geographical proximity may have resulted in some contamination among the groups. Second, the end-of-study assessment of TBAs’ knowledge of HIV and PMTCT was performed via phone calls due to the COVID-19 lockdown, unlike the face-to-face interviews at baseline. Although we used the same interviewers and retrained them for the purpose, the mode of interview may have impacted the findings.

## Conclusions

In conclusion, the combination of case managers and PMTCT training was more effective than training alone for TBAs in facilitating the linkage to care of HIV-positive pregnant women, although this effect did not reach statistical significance. Neither training nor the utilization of case managers had any statistically significant improvement in the knowledge of the TBAs at the end of the study. Larger-scale studies to further investigate the benefits of case manager support in facilitating the linkage to care for the prevention of mother-to-child transmission (PMTCT) of HIV are recommended.

### Supplementary Information

**Supplementary file 1.**

**Supplementary file 2.**

## Data Availability

The datasets used during the current study are available from the corresponding author upon reasonable request.

## Abbreviations

ANOVA: Analysis of variance
ARV: Antiretroviral
cART: Combined antiretroviral therapy
CD4: Cluster of differentiation 4
CI: Confidence interval
CM: Case manager
COVID-19: Coronavirus disease
HIV: Human immunodeficiency virus
HTS: HIV testing services
LGA: Local government area
MOH: Medical officer of health
MTCT: Mother to child transmission
OR: Odds ratio
PMTCT: Prevention of mother to child transmission
SDG: Sustainable development goals
TBA: Traditional birth attendant
WHO: World health organization
