# Supplementary material for: Impact of training and case manager support for traditional birth attendants in the linkage of care among HIV-positive pregnant women in Southwest Nigeria: a 3-arm cluster randomized control trial
Source: BMC Pregnancy Childbirth. 2024 Feb 21;24:153. doi: 10.1186/s12884-024-06332-2 (PMC10880323; doi:10.1186/s12884-024-06332-2)
Supplement: Supplementary file 1 [file 12884_2024_6332_MOESM1_ESM.doc]

**COLLEGE OF MEDICINE OF THE UNIVERSITY OF LAGOS**


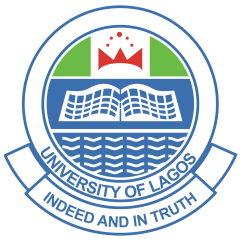


**
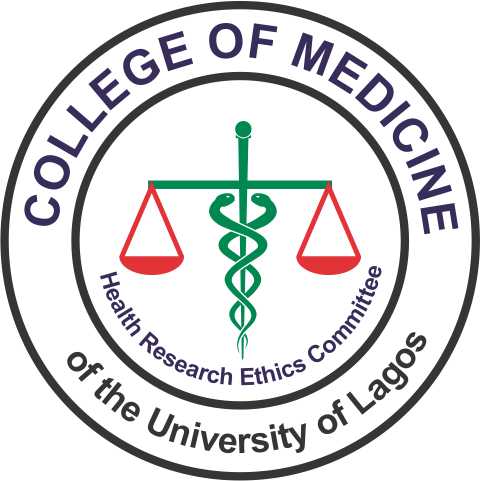
**

**HEALTH RESEARCH ETHICS COMMITTEE**

# E-mail: [hrec@cmul.edu.ng](mailto:hrec@cmul.edu.ng), Phone No: 012932526, 08028642463

#

**CMUL**

**HEALTH RESEARCH ETHICS COMMITTEE**

**FULL PROTOCOL FOR ETHICAL APPROVAL**

RESEARCH PROPOSAL

Four (4) spiral-bounded completed copies of this form should be submitted at the HREC Secretariat

***For the use of HREC Secretariat:***

*Date Received:*

*ID No:*

#### *PART I. ADMINISTRATIVE INFORMATION*

| **Please complete the required spaces.** | | | |
| --- | --- | --- | --- |
| **1.1 Name of Principal Investigator and institutional affiliation**: | | | |
| **Title**: Dr | **Surname**: Ogunyemi | | **First name**: Adedoyin |
| Name of Department / Institution/Organization Dept. of Community Health and Primary Care, CMUL.  Program in view (if applicable)  **N/A** | | | |
| Full postal address of Principal Investigator to be used for correspondence  **Dr Adedoyin Ogunyemi**  **Dept. of Community Health and Primary Care**  **College of Medicine, University of Lagos** | | | |
| Telephone: 08063068858 | | Fax: | |
| E-mail: doyinogunyemi@gmail.com | | E-mail 2: aoogunyemi@cmul.ed.ng | |
| **1.2 Title of project:** (30 words maximum) ID Number:    [LEAVE BLANK]  **The effectiveness of training models for rural traditional birth attendants in the prevention of mother to child transmission of HIV in Ogun State**  **Have you sent this protocol for review else where? Yes/No**  **If yes, please specify:** | | | |

| **Sponsor (s) of the Investigation: BRAINS** |
| --- |

| Signature and date of Principal Investigator:  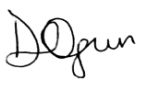 07/03/19 |
| --- |

1. Summary of study

Mother to child transmission (MTCT) accounts for 90% of all new HIV infections in Nigerian children and for about 30% of the global burden.^1,2^ In developed countries, MTCT rates have been reduced to <2%, yet high transmission rates persist in sub-Saharan Africa due to low rates (11.7% and 17.1%) of access to HIV testing and antiretroviral prophylaxis respectively.^3,4^ Studies found that about 60% of births occurred in the home of TBAs (Traditional birth attendants) especially in rural Nigeria.^5,6^ Since facility antenatal care is the main vehicle being used for PMTCT services in Nigeria, missed opportunities abound.^7,8^ TBAs are defined by WHO as “a person who assists the mother during childbirth and initially acquired her skills by delivering babies herself or through an apprenticeship to other TBAs”.^9^ Despite the vast role TBAs play in the health system, they are perceived to contribute to the high maternal mortality ratio in Nigeria due to some adverse practices.^10^

Previous studies have reported that TBAs involvement in HIV prevention and PMTCT programs could reduce HIV infant infections.^8,11,12^ This involvement has ranged from training in HIV testing and counseling, some PMTCT services including administering Nevirapine to newborns.^13^ The main barriers encountered by TBAs involvement in PMTCT service cascade are the linkage to care and the follow-up of identified HIV-positive pregnant woman and their exposed newborn according to the current guidelines.^14,15^ This includes that the woman agrees and adheres to ARV prophylaxis and commits to the same for the newborn to avert MTCT. To close this gap, community support services such as the use of case managers have been successful in community ART services.^16,17^ The case manager makes referrals, coordinates care with providers and specialists, and manages the exchange of information between providers and human services organizations.^18^ This research aims to determine the effectiveness of combining TBAs PMTCT training with HIV-case managers to complete the community PMTCT cascade.

*Methodology*: This will be a 3-arm cluster-randomized control trial to compare the effectiveness of two training models. TBAs who receive PMTCT training only (PT), and a second arm that receive PMTCT training and case manager support (PT/CM). This is a study that will involve TBAs in two LGAs in Ogun State and trained case managers. A mixed method will be used to explore factors that affect implementation. Data tools will include baseline questionnaires and TBA logbooks containing steps in the PMTCT cascade. The data will be analyzed using SPSS version 23. Ethical approval will be obtained from Health Research and Ethics Committee, College of Medicine, Idi-araba and informed consent from the study participants. The duration of this study will be 12 months.

*Expected results:* To examine the effectiveness of two training models for PMTCT intervention using the cascade. Additionally, data will be collected on implementation outcomes such as feasibility and acceptability that assesses how the training models work in practice. We will provide preliminary data that will help to design a large-scale dissemination study. Finally, better understanding of training needs and preferences among TBAs will be helpful for planning future interventions.

1. Introduction/ Background information (citing relevant criteria)

Mother to child transmission (MTCT) accounts for 90% of all new HIV infections in Nigerian children and the country accounts for about 30% of the global burden of MTCT.^1,2^ Over 60% of births occurred in the home of TBAs in rural Nigeria.^6^ In Nigeria, the main vehicle for PMTCT services are the health facilities leading to missed opportunities.^7,8^ Studies have found that the inclusion of TBAs in the PMTCT process reduces maternal to child transmission of HIV.^8,11,12^

1. Rationale/ justification of study (citing relevant criteria)

Most of these studies done among TBAs in Nigeria have focused on training them to identify HIV-positive pregnant women and refer to ART sites but loss to follow up has been reported.^14,15^ The contribution of the proposed research is expected to identify an effective training model that incorporates community resources to ensure the complete spectrum of the PMTCT cascade for prong 3 (prevention of HIV from a woman living with HIV to her infant) is followed. This contribution is significant because it has the potential to strengthen the role of TBAs in achieving other prongs in the PMTCT cascade as it allows for a feedback mechanism between the woman and the TBA. The proposed research aligns with Sustainable Development Goal three (SDG 3) target two which is to end preventable deaths of newborns and children under-5 years of age and under-5 mortality.^19^

1. Study objectives

1. To compare the effectiveness of PMTCT training (only) of TBAs and combining PMTCT training and community support services (case managers) in implementing the PMTCT cascade.

2. Use mixed methods to identify actionable factors within the implementation context that influence the effectiveness of the intervention delivered by two models.

1. Hypotheses
2. We hypothesize that both models will produce positive changes (increased access to HCTs, ARTs and ARVs) in PMTCT, compared to the control group. However, secondary outcomes of specific practices and process outcomes, such as cost, will vary.
3. We hypothesize that programs with more supportive implementation contexts will more effectively implement PMTCT than programs with less supportive contexts. Qualitative data will help explain how aspects of the implementation context influence effective implementation of each model in greater depth
4. Methods (To include study design, determination of sample size, exclusion/ Inclusion criteria, analyses of data etc)

This will be a 3-arm cluster randomized control trial (unblinded) to compare the effectiveness of two interventions. TBAs who receive **PMTCT training only (PT;Arm 1),** and a second group that receive **PMTCT training and case manager support (PT/CM;Arm 2)**. The third group will serve as the control but will also receive PMTCT training after the study. The study population will include registered TBAs in Ifo and Ado-Odo/Ota Local Government Area (LGA) in Ogun State. The prevalence of HIV among pregnant women in Ogun State is 2.9%.^20^ Eligibility criteria will include registered TBA’s not less than 18 years, who recorded two or more births in their birthing homes in previous month, who have proper record documentation and have not received PMTCT training previously. The research team will work with the medical officer of health (MOH), head of TBA associations and the LGA staff in determining TBAs who meet the eligibility criteria. After such list has been compiled, all eligible TBAs will be invited to participate in the study by sending text messages to their phones.

The MOH will also help select 12 staff who have some health training background from the LGA. Eight of them will serve as research assistants and four as case managers. Four research assistants will be assigned to the control, another four research assistants will be assigned to the PT;Arm 1 while the four case managers will be assigned to the PT/CM;Arm 2. The TBA informs the case manager (CM) when a pregnant woman is tests HIV positive. The CM links up with the HIV-positive pregnant woman and takes her to the designated PMTCT centre and manages the exchange of information between PMTCT provider and the pregnant woman with the objective of ensuring that the pregnant woman and her baby complete the PMTCT cascade of care. The research assistants and case managers will receive a 2-day training from a PMTCT expert on the natural history of HIV, antenatal testing, methods of MTCT of HIV, HAART in pregnancy and infant testing. Furthermore, the four case managers only will be trained on the strategies for linkage and retention in care using the national guidelines for HIV prevention, treatment and care.^21^ The 8 research assistants will be assigned to TBAs in the control and PT;Arm1 groups to collect data from the TBA log registers on PMTCT at the end of each month. The four case managers will be assigned to TBAs in the PT/CM;Arm2 to support PMTCT linkage services of HIV-positive pregnant women and collect their data at the end of each month for the period of six months.

The TBAs in Ifo and Ado-Odo/Ota Local Government Areas belonged to three different associations and clustered as such. In each cluster group, the research assistants will meet all eligible respondents to give information about the study and administer consent forms and to obtain their permission to be involved in the study. After consent, baseline questionnaire will be interviewer-administered to TBAs in each group to determine baseline knowledge and PMTCT practices. After the baseline survey in the three groups, the head of each of the 3 TBA groups will be invited by the principal investigator to a meeting. At the meeting, each TBA head will pick an opaque sealed envelope from a pool that contains an allocation card that indicates the arm the TBAs in the associations has been assigned to. One group will be randomized into the PT group, another group to the PT/CM group and the third group of TBAs will serve as controls. Meetings will be held with TBAs in each of the intervention and control groups. At the end of each meeting, an equal proportion of TBAs will be assigned to each research assistant for the ‘training only’ and control groups. Similarly, the case managers will also be assigned to TBAs in the PT/CM group. The research assistants will introduce themselves to the TBAs, collect the TBA birthing home details and distribute TBA logs to each TBA. Additionally, the CMs will give their contact details how they can be reached to the TBA for ease of contact when they identify a pregnant woman who is HIV-positive. for introductions and further details while the other two groups will meet with RAs. The TBAs will be given the details of six PMTCT centres within the LGAs where referrals should be made in the training only and control groups while the PT/CM group will be asked to contact their case managers. Also, coloured plastic bottle covers were given to the TBAs for positive pregnant women to hand over at the referral PMTCT centre.

TBAs who meet the eligibility criteria within the two LGAs will be enrolled in each arm to detect the difference and to achieve a power of 0.80 for a significant difference of 0.05 (2 sided).

The formula for calculating sample size of randomised controlled trial is


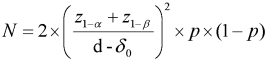


Where N=size per group;

p=the response rate of standard treatment group = prevalence of practice mother to child transmission among TBAs = 0.43^27^

z_x_= the standard normal deviate for a one or two sided x;

d= the real difference between two treatment effect; δ_0_= a clinically acceptable margin;

d- δ_0_ = effect size. Assume an effect size of 15% (= 0.15)

z_β =_ 0.845 (for power of 80%) and z_α_ = 1.645 (At level of significance of 0.05)

N = 2 x (0.845 x 1.645/0.15)^2^ x 0.43 x (1-0.57) = 42

i.e Minimum of 42 TBAs per arm

Intervention: The PT group of TBAs will receive PMTCT training only. This will include didactic modules that will take place for 2 days. It will also include HIV test counseling, information on ART referral centres, patient confidentiality and the use of the TBA logbook, as this will be a vital instrument for monitoring and evaluation. The PT/CM group will receive the above training and case manager (CM) support. The case manager is assigned to the TBA and will visit the TBAs in their homes fortnightly to review the TBA logs. The TBA will inform them of all new patients and newly detected HIV positive pregnant women, CM will facilitate linkage support to pre-determined PMTCT sites in the LGA. The training will be conducted in the local language (Yoruba). All three groups will be assigned RAs (blinded) who will have copies of the TBA logs and visit fortnightly to fill in the steps in PMTCT cascade per client seen by all the TBA throughout the period of study.

At the end of the study, one focus group discussion of 10 TBAs drawn from each of the TBA associations groups and one-on-one interviews with the case managers will be done. The aim is to understand the most influential factors in the successful implementation of PMTCT. This finding would allow practitioners choose the better model that suits their setting. All the TBAs who participate in the study will be provided with delivery kits as incentives for their clients. The components of the kit are meant to promote WHO hygienic birth practices called clean delivery and cord care.^22^ At baseline and at the end of the study period, PMTCT knowledge and practices will be assessed for all the TBAs using a pretested structured questionnaire in Yoruba language. Open ended questions will be utilized for the qualitative data. TBA logs will contain the following steps in the cascade according to the WHO guidelines.^23,24,25,26^ (See Appendix A)

Appendix A

| Women attend 1^st^ ANC visit | Women accept HIV testing | HIV-ve women retested at labour | HIV +ve women identified | HIV +ve women initiated on ART | HIV +ve women retained on ART at delivery* | Exposed infant initiated on ARV prophylaxis | Exposed infant tested for HIV at 6-8 weeks | HIV infected infant identified at 6-8 weeks |
| --- | --- | --- | --- | --- | --- | --- | --- | --- |
|  |  |  |  |  |  |  |  |  |

* For the purpose of this study instead of 12 and 24 months

Data Analysis: The data will be analyzed using SPSS version 23. Frequencies and proportions of participant’s baseline and follow-up knowledge and practices will be computed. Chi square will be used to compare proportions between the study arms. ANOVA and logistic regression for multivariate analyses will be used to adjust for significant differences identified at baseline between intervention and the usual practice arm in the outcome analyses. The primary outcome is the proportion of women who complete the PMTCT cascade i.e (HIV exposed infant testing at 6-8 weeks  among positive pregnant women) OR (HIV-ve women retested at term/labour for negative pregnant women) while the secondary outcomes will be to compare study arms with respect to degree of changes in knowledge, attitude, PMTCT practices and efficacy. Additionally, a cost analysis of the case manager intervention will examine utilization data and develop predictive models of MTCT of HIV prevented and cost savings. The level of significance will be set at p<0.05. Qualitative data will be analyzed and arranged thematically. The period of duration for the study will be 12 months.

1. Ethical consideration. Consent documentation (Translation to local languages where applicable) and Patient information leaflet (where applicable)

Participants will be asked to sign a written informed consent to be included in the study after a thorough explanation of the procedures, risks and benefits of the study. Confidentiality will be ensured by use of identification codes only.

1. Potential value of results (outcome of study)

The study will improve the knowledge and practices of the TBAs in preventing MTCT of HIV. It will improve maternal and child outcomes and improve the safety practices of the TBAs. It is expected to determine the effectiveness of the two training models and the feasibility and acceptability in practice. Overall it addresses SDG 3, focuses on the HIV-vulnerability gap among women and allows for planning future prevention interventions for women and children.

1. List of Investigators. Investigator specialty and collaborators (Attach CVs of investigators not more than 3 pages, letter of collaboration / supervision etc.)

Dr Adedoyin Ogunyemi- **PI**

Dept. of Community Health and Primary Care,

College of Medicine, University of Lagos

Dr B.O Okusanya- **Collaborator**

Dept. of Obstetrics and Gyneacology,

College of Medicine, University of Lagos

Dr K.A Odeyemi - **Mentor**

Dept. of Community Health and Primary Care,

College of Medicine, University of Lagos

1. Time – frame

One year

1. Funding

BRAINS Year 4 Grant

Here are some suggestions overall:

1. I would suggest though that you describe the training that you gave the birth attendants in each arm with more detail.  and can you please describe in more detail the case management that is done?

the more details you add to this protocol, the better-

because you will take the protocol and turn it into both the background and the methods for your manuscript that you will try to get published with the results when you have finished the trial.

1. Also why is your sample size 80 in each arm? I wasn't clear on your power calculation and how you got to this sample size?
2. It is also not clear how many people you are going to interview after the study.

How are the study operations going?

1. References
2. National Agency for the Control of AIDS. FACT SHEET: Prevention of Mother to Child Transmission (PMTCT), 2016. Available at https://naca.gov.ng/fact-sheet-prevention-mother-child-transmission-pmtct-2016/. Acces**s**ed Sep 30 2018.
3. World Health Organization. Towards universal access: scaling up priority HIV/AIDS interventions in the health sector.2010. WHO, Geneva.
4. Newell ML.  Current issues in the prevention of mother-to-child transmission of HIV-1 infection. Trans R Soc Trop Med Hyg. 2006. 100:1-5.
5. National Agency for the Control of AIDS (2012) Nigeria Global AIDS Response Country Progress Report. NACA, Abuja.
6. Nigeria Demographic and Health Survey 2013. National Population Commission Federal Republic of Nigeria Abuja, Nigeria. Rockville, Maryland, USA: ICF International; 2014. p. 104-10. Available from: https://www.dhsprogram.com/pubs/pdf/fr293/fr293.pdf
7. Ebuehi OM, Akintujoye IA.  Perception and utilization of traditional birth attendants by pregnant women attending primary health care clinics in a rural Local Government Area in Ogun State, Nigeria. 2012.Int J Women Health 4: 25-34.
8. Chizoba AE, Pharr JR, OOdo G, Ezeobi E, Ilozumba J, Egharevba J, Ezeanolue EE and Nwatu A. Increasing HIV testing among pregnant women in Nigeria. Evaluating traditional birth attendants and primary health care (Tap-In) model. AIDS Care 2017; 29(9):1094-1098.
9. Muzyamba C, Groot W, Tomini SM and Pavlava M. The usefulness of traditional birth attendants to women living with HIV in resource-poor settings: the case of Mfuwe, Zambia. Tropical Medicine and Health (2017) 45:37 DOI 10.1186/s41182-017-0076-3
10. World Health Organization. Traditional Birth Attendants: A Joint WHO/UNFPA/UNICEF Statement. Geneva: World Health Organization; 1992. p.18.
11. Ofili AN, Okojie OH (2005) Assessment of the role of traditional birth attendants in maternal health care in Oredo Local Government Area, Edo State, Nigeria. J of Community Medicine and Primary Health Care 17:55-60.
12. Gloria H, Kabondo C, Tembo T, Zimba C, Kamanga E, Mofolo I, Bulla B, Sellers C, Nakanga RC, Lee C, Martinson F, Hoffman I , Horst C and Hosseinipour MC. Evaluating the benefits of incorporating traditional birth attendants in HIV Prevention of Mother to Child Transmission service delivery in Lilongwe, Malawi. African Journal of Reproductive Health March 2014; 18(1):27
13. Che Chi P and Urdal H. The evolving role of traditional birth attendants in maternal health in post-conflict Africa: A qualitative study of Burundi and northern Uganda. SAGE Open Medicine Volume 6: 1–9
14. Gorman DA, Nyirenda LJ, and Theobald SJ. Prevention of mother-to-child transmission of HIV infection: Views and perceptions about swallowing nevirapine in rural Lilongwe, Malawi. BMC Public Health 2010, 10:354
15. Anígilájé EA, Ageda BR and Nweke NO. Barriers to uptake of prevention of mother-to-child transmission of HIV services among mothers of vertically infected HIV-seropositive infants in Makurdi, Nigeria. Patient Preference and Adherence 2016:10 57–72
16. Okoli JC and Lansdown GE.Barriers to successful implementation of prevention-of-mother-to-child-transmission (PMTCT) of HIV programmes in Malawi and Nigeria: a critical literature review study. Pan African Medical Journal. 2014; 19:154 doi:10.11604/pamj.2014.19.154.4225
17. FHI 360. SUCCESS STORY Improving access to HIV testing, care and treatment for key populations through an existing national health system: Lessons from Ghana. Available at https://www.fhi360.org/sites/default/files/media/documents/linkages-success-stories-ghana-lessons-june-2016.pdf. Accessed 1^st^ Oct 2018
18. UNAIDS. Promising practices in community engagement for elimination of new HIV infections among children by 2015 and keeping their mothers alive. 2012 http://www.unaids.org/sites/default/files/media_asset/20120628_JC2281_PromisingPracticesCommunityEngagements_en_0.pdf
19. Rural health information hub. Case Management and patient navigation https://www.ruralhealthinfo.org/toolkits/hiv-aids/2/manage/case-management
20. World Health Organization. SDG3: Ensure healthy lives and promote wellbeing for all at all ages. Available at http://www.who.int/sdg/targets/en/. Accessed 30^th^ Sep 2018.
21. Federal Ministry of Health. Federal Republic of Nigeria National HIV and AIDS Strategic Framework 2017-2021http://www.sfhnigeria.org/wp-content/uploads/2018/05/National-Strategic-frame-work-Final-28th-May-2018.pdf
22. Federal Ministry of Health. National guidelines for HIV prevention, treatment and care. 2016. Available at <http://apps.who.int/medicinedocs/documents/s23252en/s23252en.pdf>. Accessed on 30^th^ Sep 2018.
23. World Health Organization. WHO Safe Childbirth Checklist Implementation Guide Improving the quality of facility-based delivery for mothers and newborns. Available at http://apps.who.int/iris/bitstream/handle/10665/199177/9789241549455_eng.pdf;jsessionid=C5C7643EF375B6447370D53FC1E5EF76?sequence=1. Accessed 10^th^ September 2018
24. World Health Organization. Antiretroviral Drugs for Treating Pregnant Women and Preventing HIV Infection in Infants: Recommendations for a Public Health Approach. 2010 Ver. Geneva, Switzerland: World Health Organization; 2010.
25. World Health Organization. WHO Recommendations on the Diagnosis of HIV Infection in Infants and Children. Geneva, Switzerland: World Health Organization; 2010.
26. World Health Organization. Consolidated Guidelines on the Use of Antiretroviral Drugs for Treating and Preventing HIV Infection. Recommendations for a Public Health Approach. Geneva, Switzerland: World Health Organization; 2013.
27. World Health Organization. Guideline on When to Start Antiretroviral Therapy and on Pre-exposure Prophylaxis for HIV. Geneva, Switzerland: World Health Organization; 2015
28. Odeyemi K, Balogun M. Knowledge and Practice of prevention-of-mother-to-child-transmission (PMTCT) of HIV among TBA in Lagos State, Nigeria. PanAfrican Medical Journal. 2010. 5-12.
