# Supplementary material for: Impact of training and case manager support for traditional birth attendants in the linkage of care among HIV-positive pregnant women in Southwest Nigeria: a 3-arm cluster randomized control trial
Source: BMC Pregnancy Childbirth. 2024 Feb 21;24:153. doi: 10.1186/s12884-024-06332-2 (PMC10880323; doi:10.1186/s12884-024-06332-2)
Supplement: Supplementary file 2 [file 12884_2024_6332_MOESM2_ESM.docx]

S1 Appendix

| Date Pregnant woman attends 1^st^ ANC visit | Date Pregnant woman offered HIV testing | Test result received (+ve/-ve) | Date HIV +ve women referred to PMTCT centre | Name of PMTCT centre | HIV-ve women retested at term | Test result received (+ve/-ve) | Date HIV +ve women presented at the PMTCT centre |
| --- | --- | --- | --- | --- | --- | --- | --- |
|  |  |  |  |  |  |  |  |
